# Increased purchases of prescription medicines in offspring of women with type 1 diabetes: a Finnish register-based cohort study between 1995 and 2018

**DOI:** 10.1080/07853890.2024.2412283

**Published:** 2024-10-21

**Authors:** Cedric A. Korpijaakko, Johan G. Eriksson, Hannu Kautiainen, Miira M. Klemetti, Merja K. Laine

**Affiliations:** a Department of General Practice and Primary Health Care, University of Helsinki and Helsinki University Hospital, Helsinki, Finland; b Folkhälsan Research Center, Helsinki, Finland; c Yong Loo Lin School of Medicine, Human Potential Translational Research Programme and Department of Obstetrics and Gynecology, National University Singapore, Singapore, Singapore; d Singapore Institute for Clinical Sciences (SICS), Agency for Science, Technology and Research (A*STAR), Singapore, Singapore; e Primary Health Care Unit, Kuopio University Hospital, Kuopio, Finland; f Department of Obstetrics and Gynecology, Helsinki University Hospital and University of Helsinki, Helsinki, Finland; g Department of Medical and Clinical Genetics, University of Helsinki, Helsinki, Finland

**Keywords:** Offspring, type 1 diabetes, pregnancy, morbidity, prescription medicines

## Abstract

**Objective:**

This study aimed to assess whether *in utero* exposure to hyperglycemia influences prescription medicine purchases in the offspring of women with type 1 diabetes (exposed offspring).

**Patients/materials and methods:**

We identified all singleton exposed offspring born in the hospital district of Helsinki and Uusimaa, Finland, between 1988 and 2011 from the Finnish Medical Birth Register, maintained by the Finnish Institute for Health and Welfare. For each exposed offspring, we obtained five age- and province-matched offspring of women without diabetes (reference offspring), from the Finnish Medical Birth Register. By combining data from three national registers, this longitudinal cohort study assessed prescription medicine purchases in exposed offspring (*n* = 1,725) and reference offspring (*n* = 8,755) from seven to thirty years of age. Prescription medicine purchases were grouped according to the Anatomical Therapeutic Chemical (ATC) classification system.

**Results:**

Between 1995 and 2018, a total of 211,490 prescription medicines were purchased. After a median follow-up of 10.9 (interquartile range 4.9,17.6) years, we observed higher incidence risk ratios (IRR) of prescription medicine purchases for several ATC main groups in exposed offspring compared to reference offspring, with the highest IRR of 4.06 (95% CI: 2.78 to 5.94) for medicines affecting metabolism (e.g. diabetes medicines).

**Conclusion:**

Our findings suggest that exposed offspring purchase more reimbursable prescription medicines than reference offspring from age seven to thirty years. More research is needed to examine the effects of intrauterine exposure to hyperglycemia on long-term health in offspring.

## KEY MESSAGES

- It has been suggested that exposure to maternal diabetes *in utero* may program health and disease in the adulthood of the offspring.
- By combining several large national registers, this longitudinal cohort study observed a significant difference in purchases of several prescription medicines, a proxy for morbidity, from age seven years onward in the offspring of women with type 1 diabetes compared to the offspring of women without diabetes.
- Our findings suggest that exposure to type 1 diabetes *in utero* is associated with increased morbidity that extends beyond childhood into adulthood.

## Introduction

According to a recent meta-analysis by the International Diabetes Federation, the global prevalence of pregestational diabetes (i.e. type 1 and type 2 diabetes) in pregnancy has doubled during 1990–2020 [1]. Maternal type 1 diabetes is a significant risk factor for adverse perinatal outcomes; for example, preterm delivery, fetal macrosomia, and neonatal intensive care unit (NICU) admissions are frequently occurring outcomes in offspring born to women with type 1 diabetes (exposed offspring) [2–4]. It is worth noting that adverse birth outcomes are not merely limited to short-term health; rather, early life insults have been observed to alter the metabolic profile beyond infancy and extend into adulthood [5,6].

Previous clinical studies suggest that intrauterine exposure to type 1 diabetes increases offspring’s risk of developing obesity and dysmetabolic traits in adulthood [7,8]. This association is also supported by results from long-term epidemiological studies indicating that offspring of women with pregestational diabetes have an increased risk of developing early-onset cardiovascular disease (CVD) and type 2 diabetes [9,10]. In addition to unfavorable metabolic consequences, in utero exposure to pregestational diabetes have also been reported to carry a higher risk of developing asthma and psychiatric diseases [11,12]. It is noteworthy that type 1 diabetes is strongly influenced by genetic predisposition, which, due to genetic overlap, can contribute to the development of diseases with high genetic susceptibility [13,14]. That said, it is surprising that long-term morbidity in exposed offspring have been assessed in only a few longitudinal studies [10,15–16]. More specifically, previous studies have examined hospital admissions, clinical diagnoses, mortality rates, and overall use of medication in offspring of type 1 diabetes women and offspring of women without diabetes with somewhat contradictive findings [10,15–16]. We conducted a literature search from PubMed/Medline, Scopus, and Science Direct, and found that only one previous study has explored morbidity from the perspective of prescription medicine purchases in exposed offspring [15].

The incidence of type 1 diabetes displays a significant geographical variability; with Finland reporting the highest incidence of childhood type 1 diabetes globally [17]. In Finland, national health registers are considered to be of high quality and provide a great opportunity for conducting register-based studies [18]. We hypothesize that being born to a mother with type 1 diabetes may impact the offspring’s long-term health and thereby influence prescription medicine purchases. Therefore, this present study aims to investigate whether exposure to type 1 diabetes during pregnancy influences prescription medicine purchases in the offspring from the age of seven to 30 years of age, by combining data from national registers.

## Patients/materials and methods

This longitudinal cohort study was conducted in the hospital districts of Helsinki and Uusimaa, Finland. The hospital district of Helsinki and Uusimaa is the largest healthcare provider in Finland, which consists of five distinct hospital areas and arranges specialist care for the local residents of southern Finland (∼2.2 million).

### Study participants

**Figure 1.**
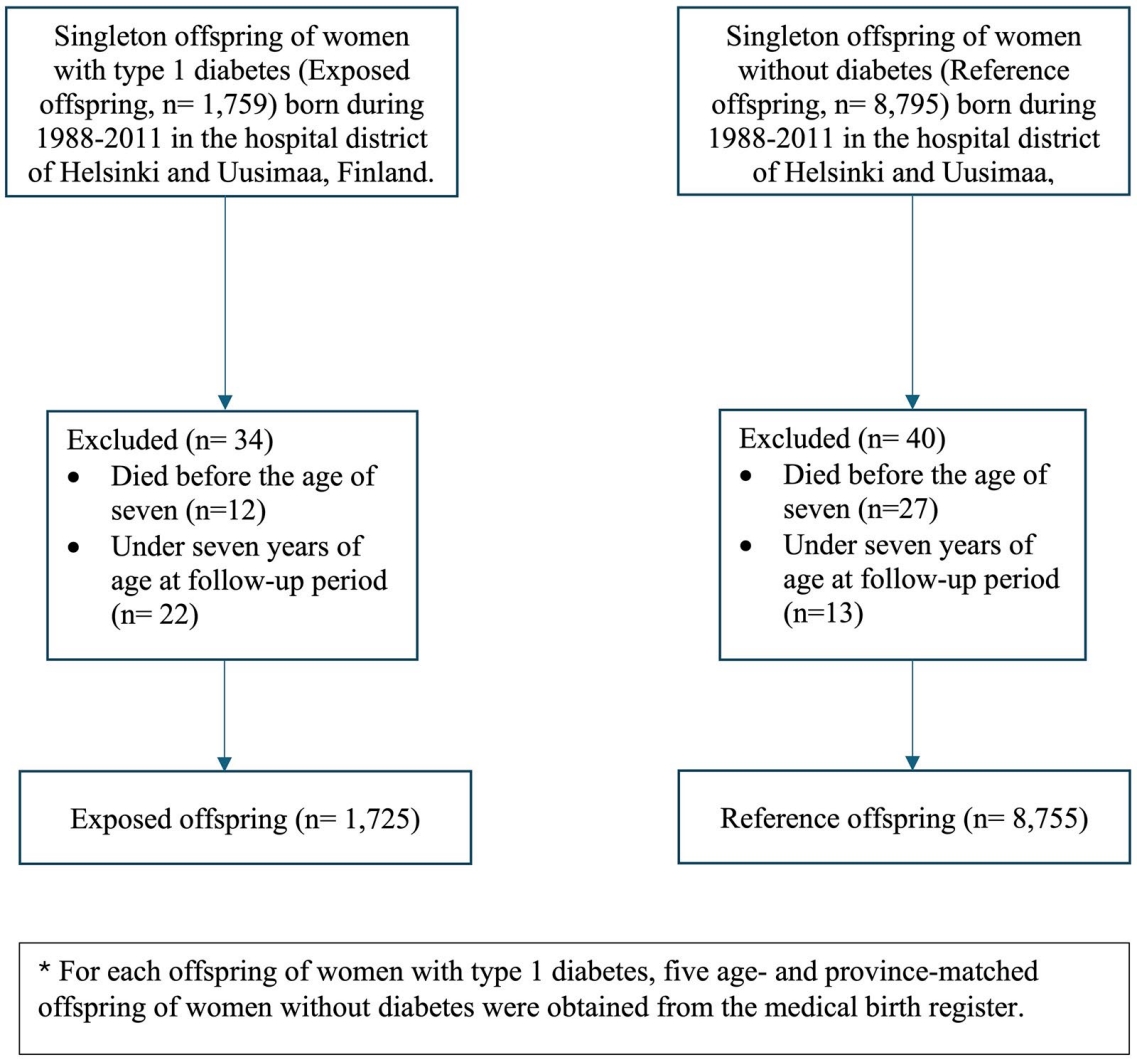
Flowchart for the study population.

The study cohort (*N* = 10,480) consisted of singleton exposed offspring followed-up from the age of seven years onwards (*n* = 1,725) and their age- and province-matched reference offspring, i.e. singleton offspring born to women without diabetes (reference offspring, *n* = 8,755). The flowchart of the study population is presented in Figure 1. The minimum age of the study participants was set to seven years in order to get a more accurate picture of the long-term health outcomes.

### Data on maternal characteristics

Data on maternal characteristics were obtained from the Medical Birth Register maintained by the Finnish Institute for Health and Welfare. The Medical Birth Register – established in 1987 – receives data on all live births and stillbirths from 22 gestational weeks onwards or neonatal weight of 500 grams from all Finnish delivery hospitals. From the Medical Birth Register, we obtained the following data on maternal characteristics: age at delivery, weight, height, smoking, mode of delivery, and the presence of diabetes. Body mass index was calculated as weight (in kg) divided by height squared (m^2^). In Finland, the common clinical practice considers women with type 1 diabetes as a risk pregnancy; hence, the delivery of women with type 1 diabetes was centralized to specialist care at university hospitals. Consequently, during 1988–2011, all women with type 1 diabetes within the hospital district of Helsinki and Uusimaa gave birth at the Department of Obstetrics and Gynecology, Helsinki University Hospital.

Women without diabetes during pregnancy were defined as lacking a diabetes diagnosis in the Medical Birth Register and having no documented purchases of glucose-lowering prescription medicines. Data on prescription medicine purchases were obtained from the Prescription Register administered by the Social Insurance Institution of Finland.

### Data on offspring’s characteristics

From the Medical Birth Register, we obtained the following data on offspring’s characteristics: gestational age at birth, birth weight, birth length, the need for NICU or respirator treatment before the age of seven days, and 1-minute Apgar score. Ponderal index was calculated as birth weight (kg)/birth length (m^3^) [19]. Offspring birth weight was calculated as z-score, which is the standard deviation score and compares a child’s weight to the weight of a child of the same length and sex. Data on mortality were obtained from Statistics Finland [20].

### Data on purchased reimbursable prescription medicines

Data on purchased prescription medicines for each study participant was collected from the Prescription Register for the years 1995–2018. Since 1995, the Prescription Register has documented reimbursable prescription medicines that have been purchased from local pharmacies. The main outcome of interest was the amount of reimbursable prescription medicines that were purchased in exposed offspring. The prescription medicines were grouped in the Prescription Register according to the World Health Organization’s (WHO) Anatomical Therapeutical Chemical (ATC) classification system. In the ATC classification, all medicines are divided into a hierarchy of five levels according to each medicine’s characteristics. The main ATC groups are as follows: group A = Alimentary tract and metabolism, group B = Blood and blood forming organs, group C = Cardiovascular system, group D = Dermatologicals, group G = Genito urinary system and sex hormones, group H = Systemic hormonal preparations (excluding sex hormones and insulins), group J = Antiinfectives for systemic use, group L = Antineoplastic and immunomodulating agents, group M = Musculo-skeletal system, group N = Nervous system, group P = Antiparasitic products, insecticides, and repellents, group R = Respiratory system, group S = Sensory organs, and group V = various. The complete and in-detail understanding of the ATC classification can be assessed from WHO sources [21]. Defined Daily Dose (DDD) was calculated according to WHO’s definition, which can be interpreted as the average daily dose for a prescription medicine used for its primary indication in adults [21]. We excluded prescription medicine purchases from our data analysis that were labeled as group E = base creams, group A01 = stomatological preparations, and group V = various, as DDD cannot be calculated for these medicines.

### Statistical analyses

We used summary statistics, means with standard deviations (SD), median with interquartile range (IQR), and counts with percentage (%) for continuous and categorical variables, respectively, to describe the study population. The exposed offspring and reference offspring were compared with the t-test or bootstrap type t-test, for continuous variables, and Pearson’s chi-square test, for categorical variables. Incidence and incidence rate ratios (IRRs) of purchased and DDD per person-years were calculated using Poisson regression models or random-effects negative binomial regression models (unstructured correlation structure) with robust standard errors, as appropriate in count data. The regression model (Poisson regression model or random-effects negative binomial regression model) was selected by using chi-squared on the goodness-of-fit test. The assumptions of overdispersion in the Poisson model were tested using the Lagrange multiplier test. Stata 17.0 (StataCorp LP, College Station, TX, USA) was used for the analysis.

## Results

The characteristics of the study population are shown in Table 1. Exposed offspring showed significantly increased signs of less optimal birth anthropometry compared to reference offspring. Also, referrals to NICU and respiratory support occurred more commonly in exposed offspring than in reference offspring.

**Table 1.** Clinical characteristics of the study population.

	Exposed offspring, *n* = 1,725	Reference offspring, *n* = 8,755	*P*-value
**Neonatal outcomes**			
Boys, *n* (%)	889 (52)	4392 (50)	0.30
Gestational age at birth, weeks, mean (SD)	36.9 (2.0)	39.8 (1.7)	<0.001
<32 w, *n* (%)	*49 (3)*	*49 (1)*	
Length at birth, cm, mean (SD)			
Boys	49.6 (3.0)	50.6 (2.4)	<0.001
Girls	48.9 (3.1)	49.8 (2.3)	<0.001
Birth weight z-score, unit, mean (SD)	0.49 (1.00)	−0.10 (0.97)	<0.001
Weight at birth, g, mean (SD)			
Boys	3657 (780)	3607 (560)	0.024
Girls	3590 (755)	3471 (521)	<0.001
Ponderal index, kg/m^3^, mean (SD)			
Boys	30.0 (3.1)	27.7 (2.6)	<0.001
Girls	30.8 (6.7)	28.0 (2.6)	<0.001
Neonatal complications			
Apgar score at 1 min, mean (SD)	8.3 (1.4)	8.8 (1.1)	<0.001
<7, *n* (%)	171 (10)	288 (3)	
Cesarean section, *n* (%)	1220 (71)	2181 (25)	<0.001
Neonatal intensive care unit, *n* (%)	1128 (65)	549 (6)	<0.001
Respiratory support, *n* (%)	88 (5)	54 (1)	<0.001
**Maternal outcomes**			
Mothers, *n*	1074	8645	
Number of offspring, *n* (%)			< 0.001
One	586 (33)	8536 (97)	
Two	768 (45)	216 (2)	
Three or more	389 (31)	3 (1)	
Maternal age at delivery, years, mean (SD)	29.4 (5.1)	30.0 (5.2)	<0.001
Maternal			
Height, cm, mean (SD)	166 (6)	166 (6)	0.77
Weight, kg, mean (SD)	67.5 (11.6)	65.4 (13.2)	<0.001
Pre-pregnancy body mass index, kg/m^2^, mean (SD)	24.5 (3.9)	23.7 (4.5)	<0.001
Smoking, *n* (%)			0.21
Continuous	232 (14)	1047 (12)	
Discontinued during 1^st^ trimester	60 (4)	268 (3)	
No	1409 (83)	7198 (85)	

Exposed offspring: Offspring of women with type 1 diabetes; Reference offspring: Offspring of women without diabetes; SD: standard deviation.

**Figure 2.**
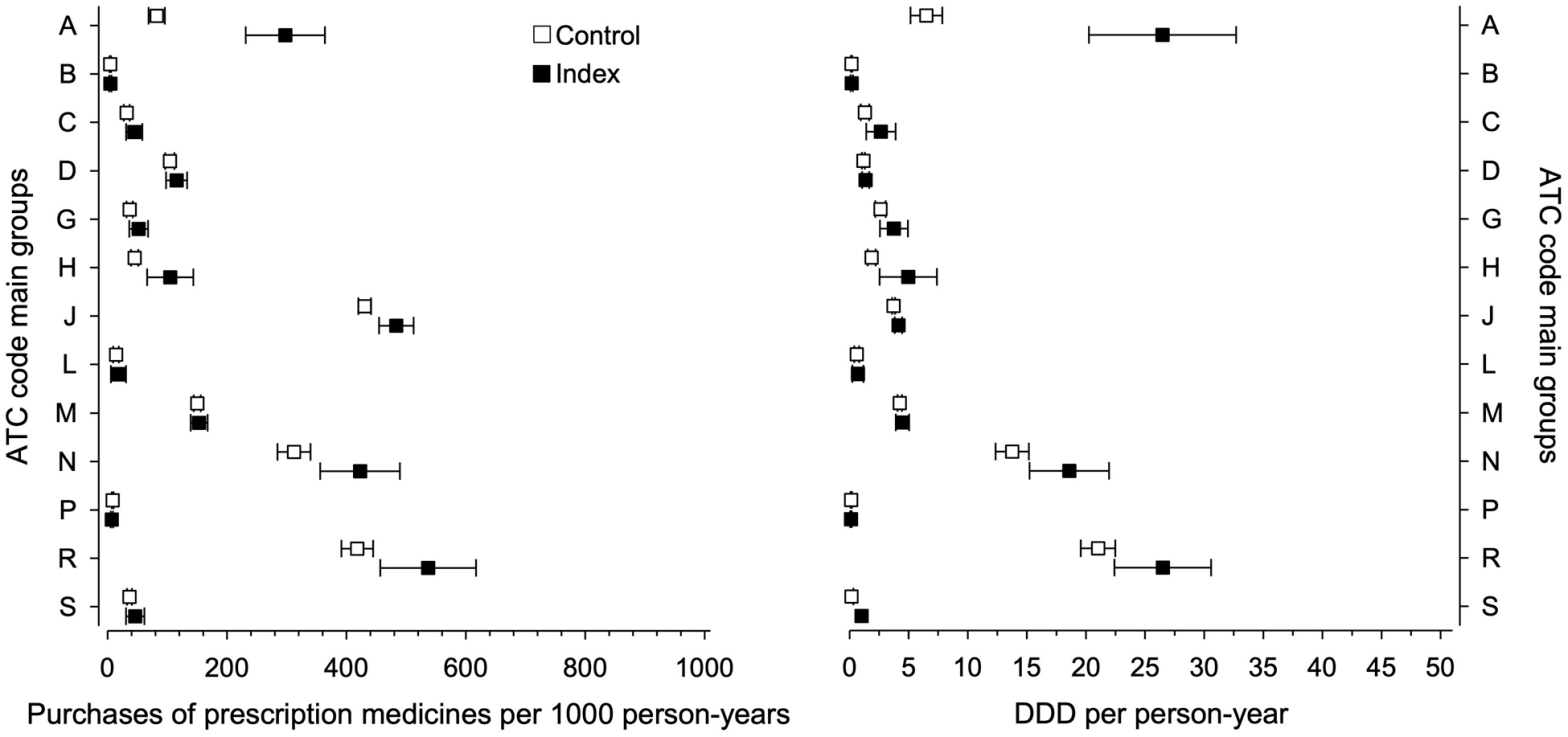
On the left (a), prescription medicine purchases are presented according to each main Anatomical Therapeutical Chemical (ATC) group by 1000 person-years in offspring of women with type 1 diabetes (exposed offspring) and in offspring of women without diabetes (reference offspring). On the right (b), prescription medicine purchases according to defined daily doses (DDD) per person-years for each main ATC group. Whiskers show 95% confidence interval. The ATC main groups: group A = Alimentary tract and metabolism, group B = Blood and blood forming organs, group C = Cardiovascular system, group D = Dermatologicals, group G = Genito urinary system and sex hormones, group H = Systemic hormonal preparations (excluding sex hormones and insulins), group J = Antiinfectives for systemic use, group L = Antineoplastic and immunomodulating agents, group M = Musculo-skeletal system, group N = Nervous system, group P = Antiparasitic products, insecticides, and repellents, group R = Respiratory system, and group S = Sensory organs.

**Figure 3.**
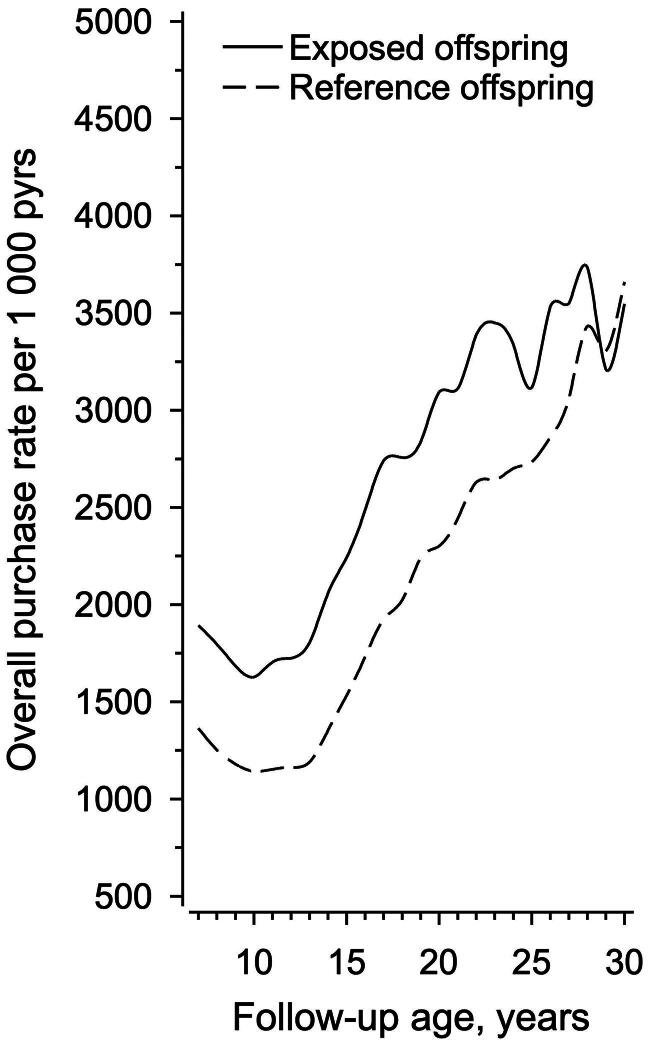
All prescription medicine purchases according to follow-up age per 1000 person-years (pyrs) in offspring of women with type 1 diabetes (exposed offspring) and in offspring of women without diabetes (reference offspring).

During a median of 10.9 (IQR 4.9,17.6) years of follow-up, 84.5% of exposed offspring purchased at least one prescription medicine; the corresponding number was 82.8% in reference offspring, respectively. A total of 211,490 prescription medicines were purchased between 1995 and 2018. The number of prescription medicine purchases according to each ATC main groups for 1000 person-years and for DDD per person-years are shown in Figure 2. In Figure 3, we present graphically the overall purchase rate of prescription medicines for both the exposed offspring and reference offspring from age 7 to 30 years of age.

Furthermore, we analyzed which specific prescription medicine groups were most frequently purchased for each ATC main group in Table 2. The most frequently purchased prescription medicine group in exposed offspring compared to reference offspring were prescription medicines used for the treatment of diabetes (A10) (IRR 5.49 [95% CI: 3.73 to 8.07]).

**Table 2.** The most frequently purchased prescription medicines within each Anatomical Therapeutical Chemical (ATC) group according to defined daily dose per person-years in offspring of women with type 1 diabetes (exposed offspring, *n* = 1,747) and in offspring of women without diabetes (reference offspring, *n* = 8,765) between 1995 and 2018.

ATC-group	Exposed offspring *n* = 1,747 Mean (SE)	Reference offspring *n* = 8,765 Mean (SE)	Incidence Risk Ratio (95% confidence interval)
Alimentary tract and metabolism (group A)			
Medicines used in diabetes (A10)	23.3 (3.1)	4.2 (0.6)	5.49 (3.73 to 8.07)
Cardiovascular system (group C)			
Agents acting on the Renin-Angiotensin system (C09)	1.2 (0.5)	0.5 (0.1)	2.43 (1.00 to 5.93)
Systemic hormonal preparations, excluding sex hormones and insulins (group H)			
Thyroid therapy (H03)	2.0 (0.5)	0.8 (0.1)	2.54 (1.43 to 4.51)
Antiinfectives for systemic use (group J)			
Antibacterials for systemic use (J01)	4.1 (0.2)	3.6 (0.1)	1.12 (1.03 to 1.22)
Nervous system (group N)			
Pscyhoanaleptics (N06)	12.2 (1.2)	8.8 (0.4)	1.39 (1.12 to 1.72)
Respiratory system (group R)			
Antihistamines for systemic use (R06)	10.8 (0.8)	9.3 (0.3)	1.16 (0.98 to 1.36)

**Figure 4.**
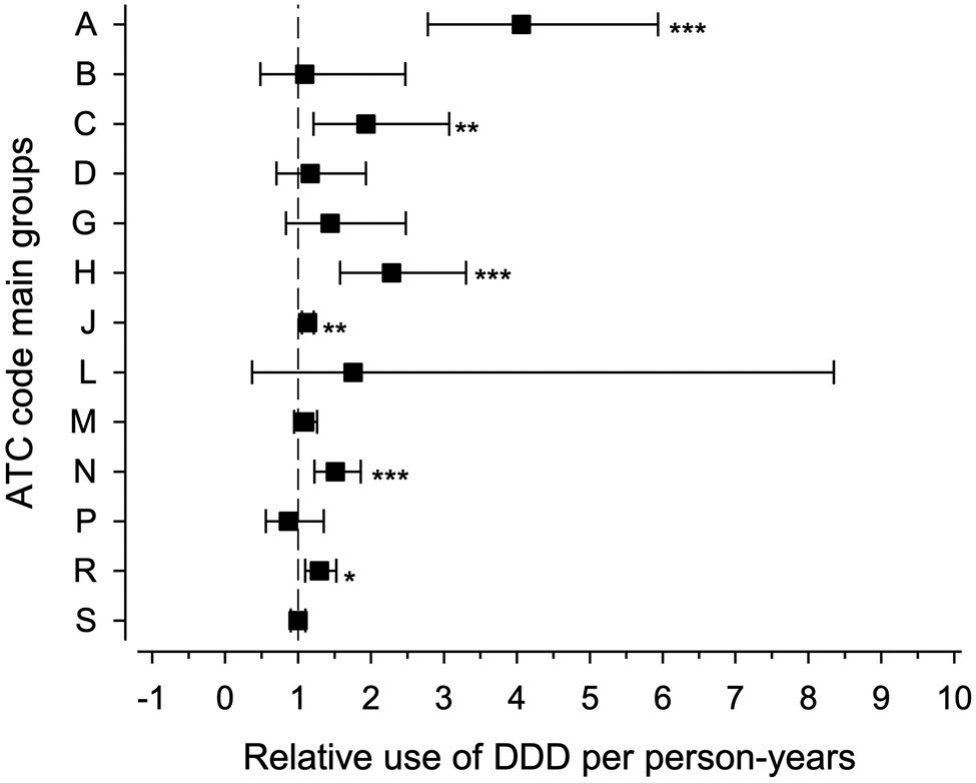
The relative use of prescription medicines between offspring of women with type 1 diabetes (exposed offspring) and offspring of women without diabetes (reference offspring) by defined daily dose (DDD) per person-years according to each main Anatomical Therapeutical Chemical (ATC). Whiskers show 95% confidence interval. *p < 0.05, ** *p* < 0.01, and ***p < 0.001. The ATC main groups: group A = Alimentary tract and metabolism, group B = Blood and blood forming organs, group C = Cardiovascular system, group D = Dermatologicals, group G = Genito urinary system and sex hormones, group H = Systemic hormonal preparations (excluding sex hormones and insulins), group J = Antiinfectives for systemic use, group L = Antineoplastic and immunomodulating agents, group M = Musculo-skeletal system, group N = Nervous system, group P = Antiparasitic products, insecticides, and repellents, group R = Respiratory system, and group S = Sensory organs.

As presented in Figure 4, we observed differences in the purchases of prescription medicines for the following main ATC groups: the alimentary tract and metabolism (group A) 4.06 (95% CI: 2.78 to 5.94), the cardiovascular system (group C) 1.93 (95% CI: 1.21 to 3.07), the systemic hormonal preparations (excluding sex hormones and insulins) (group H) 2.28 (95% CI: 1.58 to 3.30), the antiinfectives for systemic use (group J) 1.13 (95% CI:1.05 to 1.22), the nervous system (group N) 1.51 (95% CI: 1.23 to 1.86), and respiratory system (group R) 1.29 (95% CI: 1.10 to 1.52) for exposed offspring in comparison to reference offspring, respectively.

## Discussion

This longitudinal cohort study suggests that from age 7 to 30 years, exposed offspring purchase more prescription medicines compared to reference offspring. In line with Knorr et al. (2015), we found increased purchases of prescription medicines for several main ATC groups in the exposed offspring; however, there are a few differences that need to be considered [15]. Firstly, we examined prescription medicine purchases for a different offspring age range which may explain some of the observed differences in the IRR for some main ATC groups. Secondly, we also estimated prescription medicine consumption according to prescription medicine purchases to DDD per person-years (Figures 2b and 4), which is an alternative approach to estimate prescription medicine use compared to purchases.

Typically for pregnancies complicated by type 1 diabetes, we observed several adverse neonatal outcomes compared to pregnancies unaffected by diabetes [2–4]. More specifically, exposed offspring were heavier at birth with increased birth weight and increased ponderal index, regardless of sex. Also, we observed that the preterm birth rate (<32 weeks) was significantly increased in exposed offspring. Taken together, these findings suggest that unfavorable early life outcomes occur more often in exposed offspring, which might have long-term implications for the development of non-communicable diseases [5,6].

For the age range considered, we observed that the risk of purchasing prescription medicines in the treatment of diabetes (A10) was 5.49-fold in exposed offspring compared to reference offspring. This is in line with previous findings from observational studies that exposed offspring have an increased risk of both type 1 and type 2 diabetes in adulthood [7,10,22]. It is likely that there are several mechanisms involved within this association and a genetically inherited risk could provide one explanation. More specifically, Weiss et al. (2000) observed that the relative risk of developing type 1 diabetes in children born to women with type 1 diabetes was 70 times higher compared to children born to women without diabetes [22]. Also, it could be argued that a developmental origin of health and disease (DOHaD)-concept might underlie this association. The heavier birth weight that we observed in exposed offspring might convey the risk of diabetes – e.g. through epigenetic mechanisms – into adulthood [23]. This possibility is strengthened by signs of altered gene expression in the adipose tissue of young adults exposed to type 1 diabetes *in utero* [24]. Nevertheless, while the clinical significance of epigenetic modifications remains unknown, one should not overlook the impact of lifestyle behavior, which might further contribute substantially to the risk of metabolic diseases [25].

Interestingly, a new finding in our study was an IRR of 2.43-fold for purchasing agents acting on the renin-angiotensin system (C09) in exposed offspring compared to reference offspring. In fact, we observed that purchases of all prescription medicines affecting the cardiovascular system (ATC group C) was increased by 1.93-fold in exposed offspring. This result is opposite to the findings of Knorr et al. (2015), who did not observe increased purchases of CVD medications in offspring, even though they observed higher morbidity for circulatory diseases until 21 years of age [15]. Our finding is likely mediated by an interplay of several mechanisms. Firstly, substantial evidence exist that maternal hyperglycemia increases the risk of organ malformations in the offspring in a dose-dependent manner [2,4,26]. As such, individuals with mild congenital heart diseases might be overrepresented among the exposed offspring [16]. However, this seems unlikely to solely describe our finding, as longitudinal studies have observed increased risk of CVD in exposed offspring after excluding individuals with congenital heart disease [9]. On the other hand, a recent UK Biobank Cohort study observed that a unfavorable lifestyle was associated with higher risk of CVD independently of genetic risk [27]. In this case, environmental factors that could contribute to CVD might be due to developmental programming or due to behavioral differences. While a possible role for fetal programming in the development of hypertension and kidney diseases is suggested by findings from observational studies [5,23,28,29], women with type 1 diabetes might be more vigilant and use more health services for their children compared to women without diabetes.

In addition to the findings mentioned above, we observe that prescription medicine purchases were significantly increased for medicines affecting the respiratory system (ATC group R), and systemic hormonal preparations (ATC group H), which is in line with the results provided by Knorr et al. (2015) [15]. We observe that the IRR of purchasing thyroxin supplement (H03) was 2.54-fold in exposed offspring, while the IRR of purchasing respiratory medicines (ATC group R) was 1.29-fold compared to reference offspring. We believe that genetic factors would provide a feasible explanation for this relationship, especially considering the genetic overlap between type 1 diabetes, asthma, and thyroid disorders [14,30]. On the other hand, there is growing evidence for a DOHaD-concept in the development of lung diseases in offspring. Martinez et al. (2020) observed that the risk of asthma was increased in the offspring of mothers with preexisting type 2 diabetes, regardless of other important maternal confounders like asthma, smoking, and obesity [11]. Also, preterm birth has been shown to impact long-term lung function [31]. That said, considering that the adverse neonatal outcomes occurred more frequently in the offspring of women with type 1 diabetes in this cohort, exposure *in utero* to type 1 diabetes combined with postnatal insults might provide alternative pathways for the development of respiratory diseases.

We observed increased purchases of prescription medicines affecting the nervous system (ATC group N) in exposed offspring. Within this category, we specify that the most frequently purchased prescription medicines from age 7 to 30 years were psychoanaleptics (N06, i.e. antidepressants and psychostimulants or agents used for attention-deficit and/or hyperactivity disorder [ADHD]). This finding is in line with Bytoft et al. (2017), who observed that offspring of women with type 1 diabetes (mean age 17 years) had increased use of ADHD medication compared to offspring of women without diabetes [32]. While ADHD is a polygenic disorder, several environmental factors are known to increase disease susceptibility [33]. Previous studies have observed that exposure to maternal diabetes, obesity, and autoimmune diseases *in utero* increase ADHD risk, and based on our results type 1 diabetes may also increase this risk [12,34,35].

One key strength of this study is the use of several national registers providing up to 23 years of follow-up data. To date, to the best of our knowledge only one previous study has explicitly investigated morbidity in relation to prescription medicine purchases in offspring exposed to pregestational diabetes. In addition to solely examining prescription medicine purchases, we estimated prescription medicine use according to DDD per person years.

Among the limitations of the present study is the clustering of observations, which suggests that second- degree relatives are more likely to be included at higher frequencies among the exposed compared to the unexposed. This can alter the strength of observations. We do not have information on the subsequent morbidity of the mothers, such as whether the mothers in the control group developed diabetes after pregnancy. This may impact the subsequent morbidity of the offspring. Further, it is known that birth order of offspring can impact on future health [36,37]. However, we were unable to conduct subgroup analyzes to assess whether there existed sibling rank for prescription medicine purchases. Also, the use of DDD is validated only for adults, which might impact the generalizability of our findings for younger study participants. Nonetheless, for several prescription medicines, the treatment dose is similar after the age of 16. Another limitation that should be noted is that we lack data on the clinical diagnoses of the study participants. However, we interpret that reimbursable prescription medicines are prescribed primarily for clinical diseases and for symptom relief – either way, increased purchase of prescription medicines is a proxy measure of morbidity. Also, the incomplete documentation in the Medical Birth Register regarding some maternal characteristics is a limitation. Variables such as maternal obesity, gestational weight gain, glycemic control, and hypertensive disorders during different trimesters affect offspring’s outcomes; however, these factors were missing or incompletely documented in the registry, making it unachievable for more detailed data analysis. That said, the possibility remains that unmeasured confounders might be underlying some of the observed associations.

## Conclusions

In conclusion, our study finds that offspring of women with type 1 diabetes purchase more prescription medicines than offspring of women without diabetes from age seven years into adulthood. Overall, our results suggest that the influence of type 1 diabetes in pregnancy on the offspring’s health are not merely restricted to the perinatal period but might extend into later life. The increased purchase of reimbursable prescription medicines in offspring of women with type 1 diabetes are probably mediated by several different pathways. Future well-designed prospective studies are needed to assess if similar findings are identifiable in offspring of women with type 1 diabetes in young adulthood.

## Data Availability

The data presented here are not available to the public due to legislative reasons.
